# *N*′-Cyclo­hexyl­idenebenzohydrazide

**DOI:** 10.1107/S1600536809052143

**Published:** 2009-12-09

**Authors:** Xiuping Ju, Yan Qiao, Zhiqing Gao, Lingqian Kong

**Affiliations:** a Dongchang College, Liaocheng University, Liaocheng 250059, People’s Republic of China

## Abstract

In the title compound, C_13_H_16_N_2_O, the cyclo­hexane ring adopts a chair conformation. In the crystal structure, inter­molecular N—H⋯O and C—H⋯O hydrogen bonds link the mol­ecules into chains propagating in [001].

## Related literature

For related structures, see: Fun *et al.* (2008 ▶); Nie (2008 ▶); Kong *et al.* (2009 ▶); Fan & Song (2009 ▶).
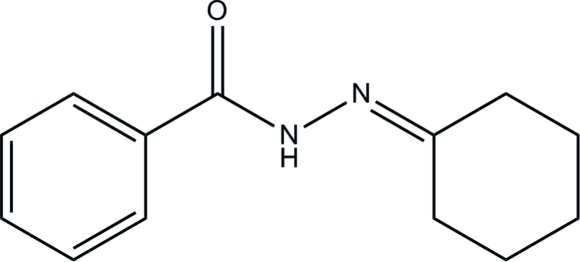


## Experimental

### 

#### Crystal data

C_13_H_16_N_2_O
*M* *_r_* = 216.28
Tetragonal, 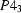

*a* = 9.4691 (11) Å
*c* = 13.8514 (15) Å
*V* = 1242.0 (2) Å^3^
*Z* = 4
Mo *K*α radiation
μ = 0.07 mm^−1^
*T* = 298 K
0.44 × 0.41 × 0.28 mm

#### Data collection

Bruker SMART APEX CCD area-detector diffractometer
Absorption correction: multi-scan (*SADABS*; Sheldrick, 1996 ▶) *T*_min_ = 0.968, *T*_max_ = 0.980
6248 measured reflections
1145 independent reflections
860 reflections with *I* > 2σ(*I*)
*R*_int_ = 0.039

#### Refinement

*R*[*F* ^2^ > 2σ(*F* ^2^)] = 0.038
*wR*(*F* ^2^) = 0.114
*S* = 1.08
1145 reflections
145 parameters
1 restraint
H-atom parameters constrained
Δρ_max_ = 0.12 e Å^−3^
Δρ_min_ = −0.13 e Å^−3^

### 

Data collection: *SMART* (Siemens, 1996 ▶); cell refinement: *SAINT* (Siemens, 1996 ▶); data reduction: *SAINT*; program(s) used to solve structure: *SHELXS97* (Sheldrick, 2008 ▶); program(s) used to refine structure: *SHELXL97* (Sheldrick, 2008 ▶); molecular graphics: *SHELXTL* (Sheldrick, 2008 ▶); software used to prepare material for publication: *SHELXTL*.

## Supplementary Material

Crystal structure: contains datablocks I, global. DOI: 10.1107/S1600536809052143/cv2665sup1.cif

Structure factors: contains datablocks I. DOI: 10.1107/S1600536809052143/cv2665Isup2.hkl

Additional supplementary materials:  crystallographic information; 3D view; checkCIF report

## Figures and Tables

**Table 1:** Hydrogen-bond geometry (Å, °)

*D*—H⋯*A*	*D*—H	H⋯*A*	*D*⋯*A*	*D*—H⋯*A*
N1—H1⋯O1^i^	0.86	2.28	3.133 (4)	172
C13—H13*B*⋯O1^i^	0.97	2.41	3.264 (5)	147
C7—H7⋯O1^i^	0.93	2.35	3.137 (5)	142

Symmetry code: (i) 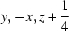.

**Fig. 1.**
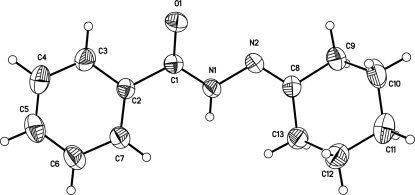
A view of (I) showing the atomic numbering scheme and 30% probability displacement ellipsoids,

## supplementary crystallographic information

### Comment

In continuation of our structural study of benzohydrazide derivatives (Kong *et al.*, 2009; Fan & Song, 2009), we present here the title compound, (I).

In (I) (Fig. 1), the bond lengths and angles are normal and comparable to those observed in the analogue compounds (Nie, 2008; Fun *et al.*, 2008). The C8=N2 bond length is 1.283 (4) ° showing the double-bond character. The dihedral angle between the benzene ring C2—C7 and the plane C1/N1/N2 is 19.0 (3) °

In the crystal structure, intermolecular N—H···O and C—H..O hydrogen bonds (Table 1) link the molecules into chains propagated in direction [001].

### Experimental

Cyclohexanone (5 mmol), benzohydrazide (5 mmol) and 10 ml of methanol were mixed in 50 ml flask. After stirring for 30 min at 373 K, the resulting mixture was recrystallized from methanol, affording the title compound as colourless crystalline solid. Elemental analysis: calculated for C_13_H_16_N_2_O: C 72.19, H 7.46, N 12.95%; found: C 72.18, H 7.25, N 12.78%.

### Refinement

All H atoms were placed in geometrically idealized positions (N—H 0.86 Å, C—H 0.93–0.97 Å) and treated as riding on their parent atoms, with *U*_iso_(H) = 1.2 *U*_eq_(C, N). In the absence of any significant anomalous scatterers in the molecule, the 1021 Friedel pairs were merged before the final refinement.

### Crystal data

**Table:**

C_13_H_16_N_2_O	*D*_x_ = 1.157 Mg m^−^^3^
*M**_r_* = 216.28	Mo *K*α radiation, λ = 0.71073 Å
Tetragonal, *P*4_3_	Cell parameters from 1861 reflections
*a* = 9.4691 (11) Å	θ = 2.6–21.7°
*c* = 13.8514 (15) Å	µ = 0.07 mm^−^^1^
*V* = 1242.0 (2) Å^3^	*T* = 298 K
*Z* = 4	Block, colourless
*F*(000) = 464	0.44 × 0.41 × 0.28 mm

### Data collection

**Table:**

Bruker SMART APEX CCD area-detector diffractometer	1145 independent reflections
Radiation source: fine-focus sealed tube	860 reflections with *I* > 2σ(*I*)
graphite	*R*_int_ = 0.039
phi and ω scans	θ_max_ = 25.0°, θ_min_ = 2.2°
Absorption correction: multi-scan (*SADABS*; Sheldrick, 1996)	*h* = −9→11
*T*_min_ = 0.968, *T*_max_ = 0.980	*k* = −9→11
6248 measured reflections	*l* = −16→15

### Refinement

**Table:**

Refinement on *F*^2^	Primary atom site location: structure-invariant direct methods
Least-squares matrix: full	Secondary atom site location: difference Fourier map
*R*[*F*^2^ > 2σ(*F*^2^)] = 0.038	Hydrogen site location: inferred from neighbouring sites
*wR*(*F*^2^) = 0.114	H-atom parameters constrained
*S* = 1.08	*w* = 1/[σ^2^(*F*_o_^2^) + (0.0557*P*)^2^ + 0.1344*P*] where *P* = (*F*_o_^2^ + 2*F*_c_^2^)/3
1145 reflections	(Δ/σ)_max_ < 0.001
145 parameters	Δρ_max_ = 0.12 e Å^−^^3^
1 restraint	Δρ_min_ = −0.13 e Å^−^^3^

### Special details

**Table:**

Geometry. All e.s.d.'s (except the e.s.d. in the dihedral angle between two l.s. planes) are estimated using the full covariance matrix. The cell e.s.d.'s are taken into account individually in the estimation of e.s.d.'s in distances, angles and torsion angles; correlations between e.s.d.'s in cell parameters are only used when they are defined by crystal symmetry. An approximate (isotropic) treatment of cell e.s.d.'s is used for estimating e.s.d.'s involving l.s. planes.
Refinement. Refinement of *F*^2^ against ALL reflections. The weighted *R*-factor *wR* and goodness of fit *S* are based on *F*^2^, conventional *R*-factors *R* are based on *F*, with *F* set to zero for negative *F*^2^. The threshold expression of *F*^2^ > σ(*F*^2^) is used only for calculating *R*-factors(gt) *etc*. and is not relevant to the choice of reflections for refinement. *R*-factors based on *F*^2^ are statistically about twice as large as those based on *F*, and *R*- factors based on ALL data will be even larger.

### Fractional atomic coordinates and isotropic or equivalent isotropic displacement parameters (Å^2^)

**Table:**

	*x*	*y*	*z*	*U*_iso_*/*U*_eq_	
N1	0.1658 (3)	0.0240 (3)	0.0959 (2)	0.0539 (7)	
H1	0.1939	−0.0292	0.1423	0.065*	
N2	0.0942 (3)	−0.0310 (3)	0.0148 (2)	0.0591 (8)	
O1	0.1613 (3)	0.2405 (2)	0.02619 (18)	0.0664 (7)	
C1	0.1877 (3)	0.1650 (3)	0.0965 (2)	0.0469 (7)	
C2	0.2512 (3)	0.2288 (3)	0.1861 (2)	0.0476 (8)	
C3	0.2322 (4)	0.3730 (4)	0.1997 (3)	0.0691 (10)	
H3	0.1785	0.4244	0.1558	0.083*	
C4	0.2925 (5)	0.4407 (5)	0.2780 (4)	0.0879 (14)	
H4	0.2800	0.5374	0.2860	0.106*	
C5	0.3704 (5)	0.3662 (5)	0.3435 (3)	0.0850 (13)	
H5	0.4090	0.4120	0.3967	0.102*	
C6	0.3921 (5)	0.2236 (5)	0.3311 (3)	0.0837 (13)	
H6	0.4468	0.1733	0.3751	0.100*	
C7	0.3317 (4)	0.1553 (4)	0.2525 (3)	0.0660 (10)	
H7	0.3456	0.0588	0.2444	0.079*	
C8	0.0953 (3)	−0.1653 (4)	0.0023 (3)	0.0571 (9)	
C9	0.0175 (4)	−0.2201 (4)	−0.0853 (3)	0.0760 (12)	
H9A	−0.0581	−0.2823	−0.0648	0.091*	
H9B	−0.0242	−0.1415	−0.1200	0.091*	
C10	0.1162 (5)	−0.2993 (5)	−0.1514 (4)	0.0919 (14)	
H10A	0.1828	−0.2336	−0.1800	0.110*	
H10B	0.0622	−0.3421	−0.2032	0.110*	
C11	0.1973 (5)	−0.4143 (5)	−0.0971 (4)	0.0898 (14)	
H11A	0.1320	−0.4873	−0.0767	0.108*	
H11B	0.2659	−0.4570	−0.1401	0.108*	
C12	0.2727 (5)	−0.3556 (4)	−0.0097 (3)	0.0824 (13)	
H12A	0.3473	−0.2927	−0.0310	0.099*	
H12B	0.3162	−0.4327	0.0255	0.099*	
C13	0.1736 (5)	−0.2748 (4)	0.0584 (3)	0.0719 (11)	
H13A	0.1073	−0.3397	0.0880	0.086*	
H13B	0.2280	−0.2301	0.1093	0.086*	

### Atomic displacement parameters (Å^2^)

**Table:**

	*U*^11^	*U*^22^	*U*^33^	*U*^12^	*U*^13^	*U*^23^
N1	0.0677 (17)	0.0506 (16)	0.0435 (16)	−0.0033 (12)	−0.0078 (14)	0.0016 (13)
N2	0.0676 (18)	0.0586 (17)	0.0510 (18)	−0.0012 (14)	−0.0130 (14)	−0.0043 (15)
O1	0.0879 (17)	0.0622 (14)	0.0491 (16)	−0.0017 (12)	−0.0084 (13)	0.0105 (13)
C1	0.0479 (17)	0.0524 (19)	0.040 (2)	0.0022 (14)	0.0066 (15)	0.0005 (16)
C2	0.0538 (18)	0.0502 (18)	0.0389 (19)	−0.0028 (15)	0.0075 (15)	−0.0034 (15)
C3	0.084 (3)	0.059 (2)	0.065 (3)	0.0076 (18)	0.001 (2)	−0.0094 (19)
C4	0.106 (3)	0.066 (3)	0.092 (4)	0.001 (2)	0.005 (3)	−0.029 (3)
C5	0.102 (3)	0.097 (3)	0.056 (3)	−0.016 (3)	−0.003 (3)	−0.024 (3)
C6	0.102 (3)	0.087 (3)	0.061 (3)	−0.008 (2)	−0.023 (2)	−0.006 (2)
C7	0.077 (2)	0.063 (2)	0.058 (2)	−0.0037 (19)	−0.011 (2)	−0.0024 (19)
C8	0.063 (2)	0.055 (2)	0.053 (2)	−0.0060 (16)	0.0021 (17)	−0.0048 (17)
C9	0.083 (3)	0.069 (2)	0.076 (3)	−0.009 (2)	−0.020 (2)	−0.013 (2)
C10	0.123 (4)	0.090 (3)	0.062 (3)	−0.016 (3)	−0.009 (3)	−0.022 (3)
C11	0.103 (3)	0.079 (3)	0.088 (4)	−0.005 (2)	0.020 (3)	−0.024 (3)
C12	0.089 (3)	0.072 (2)	0.086 (3)	0.009 (2)	0.005 (3)	−0.006 (2)
C13	0.097 (3)	0.064 (2)	0.055 (2)	0.006 (2)	0.000 (2)	0.0009 (19)

### Geometric parameters (Å, °)

**Table:**

N1—C1	1.350 (4)	C8—C13	1.493 (5)
N1—N2	1.412 (4)	C8—C9	1.511 (5)
N1—H1	0.8600	C9—C10	1.508 (6)
N2—C8	1.283 (4)	C9—H9A	0.9700
O1—C1	1.234 (4)	C9—H9B	0.9700
C1—C2	1.506 (4)	C10—C11	1.530 (7)
C2—C7	1.382 (5)	C10—H10A	0.9700
C2—C3	1.390 (4)	C10—H10B	0.9700
C3—C4	1.383 (6)	C11—C12	1.511 (6)
C3—H3	0.9300	C11—H11A	0.9700
C4—C5	1.366 (7)	C11—H11B	0.9700
C4—H4	0.9300	C12—C13	1.535 (5)
C5—C6	1.377 (6)	C12—H12A	0.9700
C5—H5	0.9300	C12—H12B	0.9700
C6—C7	1.390 (5)	C13—H13A	0.9700
C6—H6	0.9300	C13—H13B	0.9700
C7—H7	0.9300		
			
C1—N1—N2	116.3 (3)	C10—C9—H9A	109.5
C1—N1—H1	121.8	C8—C9—H9A	109.5
N2—N1—H1	121.8	C10—C9—H9B	109.5
C8—N2—N1	118.0 (3)	C8—C9—H9B	109.5
O1—C1—N1	122.5 (3)	H9A—C9—H9B	108.0
O1—C1—C2	119.9 (3)	C9—C10—C11	111.5 (4)
N1—C1—C2	117.6 (3)	C9—C10—H10A	109.3
C7—C2—C3	118.4 (3)	C11—C10—H10A	109.3
C7—C2—C1	124.5 (3)	C9—C10—H10B	109.3
C3—C2—C1	117.0 (3)	C11—C10—H10B	109.3
C4—C3—C2	120.5 (4)	H10A—C10—H10B	108.0
C4—C3—H3	119.7	C12—C11—C10	111.7 (4)
C2—C3—H3	119.7	C12—C11—H11A	109.3
C5—C4—C3	120.3 (4)	C10—C11—H11A	109.3
C5—C4—H4	119.9	C12—C11—H11B	109.3
C3—C4—H4	119.9	C10—C11—H11B	109.3
C4—C5—C6	120.3 (4)	H11A—C11—H11B	107.9
C4—C5—H5	119.8	C11—C12—C13	112.8 (3)
C6—C5—H5	119.8	C11—C12—H12A	109.0
C5—C6—C7	119.5 (4)	C13—C12—H12A	109.0
C5—C6—H6	120.3	C11—C12—H12B	109.0
C7—C6—H6	120.3	C13—C12—H12B	109.0
C2—C7—C6	120.9 (4)	H12A—C12—H12B	107.8
C2—C7—H7	119.5	C8—C13—C12	109.2 (3)
C6—C7—H7	119.5	C8—C13—H13A	109.8
N2—C8—C13	128.4 (3)	C12—C13—H13A	109.8
N2—C8—C9	116.4 (3)	C8—C13—H13B	109.8
C13—C8—C9	114.9 (3)	C12—C13—H13B	109.8
C10—C9—C8	110.9 (3)	H13A—C13—H13B	108.3

### Hydrogen-bond geometry (Å, °)

**Table:**

*D*—H···*A*	*D*—H	H···*A*	*D*···*A*	*D*—H···*A*
N1—H1···O1^i^	0.86	2.28	3.133 (4)	172
C13—H13B···O1^i^	0.97	2.41	3.264 (5)	147
C7—H7···O1^i^	0.93	2.35	3.137 (5)	142

Symmetry codes: (i) *y*, −*x*, *z*+1/4.
